# Spondylodiscitis associated with recurrent *E. coli* bacteraemia in an elderly patient: a case report highlighting uncommon pathogen presentation

**DOI:** 10.1093/bjrcr/uaaf057

**Published:** 2025-12-02

**Authors:** Yu Lelt Win, Arpan Banerjee, Inderjeet Nagra

**Affiliations:** Department of Radiology, University Hospitals Birmingham NHS Foundation Trust, Birmingham, B15 2GW, United Kingdom; Birmingham City University, Birmingham, B15 3TN, United Kingdom; Department of Radiology, The Heart of England NHS Foundation Trust, Birmingham, B9 5SS, United Kingdom; Department of Radiology, Worcestershire Acute Hospitals NHS Trust, Worcester, WR5 1JR, United Kingdom

**Keywords:** *E. coli* bacteraemia, spondylodiscitis, psoas abscess, ESBL *E. coli*, proctitis

## Abstract

Spinal infections are defined as infections within the vertebral column, intervertebral disc space, spinal canal, and surrounding soft tissues. *Mycobacterium tuberculosis* is the most prevalent pathogen worldwide, and *Staphylococcus aureus* accounts for more than half of European cases. However, *Escherichia coli* is a rare organism causing spondylodiscitis. Early diagnosis is vital for the effective management of spinal infections, as it helps prevent mortality and significant morbidity. We present a case of spondylodiscitis caused by *E. coli* bacteraemia, an atypical pathogen in this setting. The patient’s history of cancer and immunocompromised condition complicated the diagnostic process. This case emphasises the importance of thorough evaluation with imaging and highlights the role of multidisciplinary teams in overcoming diagnostic challenges.

## Clinical presentation

An 84-year-old male presented with a fever and lower back pain. He had no prior genitourinary, gastrointestinal, respiratory, or systemic symptoms. His medical history included prostate cancer treated with hormonal therapy, a bicuspid aortic valve with mixed aortic valve disease, atrial fibrillation, hypertension, and hypothyroidism. Clinical examination revealed a longstanding murmur without peripheral signs of infective endocarditis. There was no neurological deficit.

## Initial investigations and management

Initial blood tests showed an elevated white cell count of 14 000/mm^3^ and a C-reactive protein (CRP) of 209 mg/L. A chest X-ray (CXR) revealed no acute abnormalities, whilst spinal X-rays indicated degenerative disc disease without evidence of fracture, osteophytes, or metastases. Blood cultures grew *Escherichia coli* (*E. coli*); however, urine and stool cultures were negative, making the source of the *E. coli* bacteraemia unclear. The organism was susceptible to co-trimoxazole, and therefore, it was initially treated with co-trimoxazole 960 mg twice daily. One month prior, during a previous admission, the patient had also had a co-trimoxazole-sensitive isolated *E. coli* bacteraemia of unknown origin. Due to the development of acute kidney injury, the antibiotic regimen was switched to ciprofloxacin 750 mg twice daily and later reduced to 750 mg once daily because of concerns about renal dysfunction.

## Imaging

**Figure 1.**
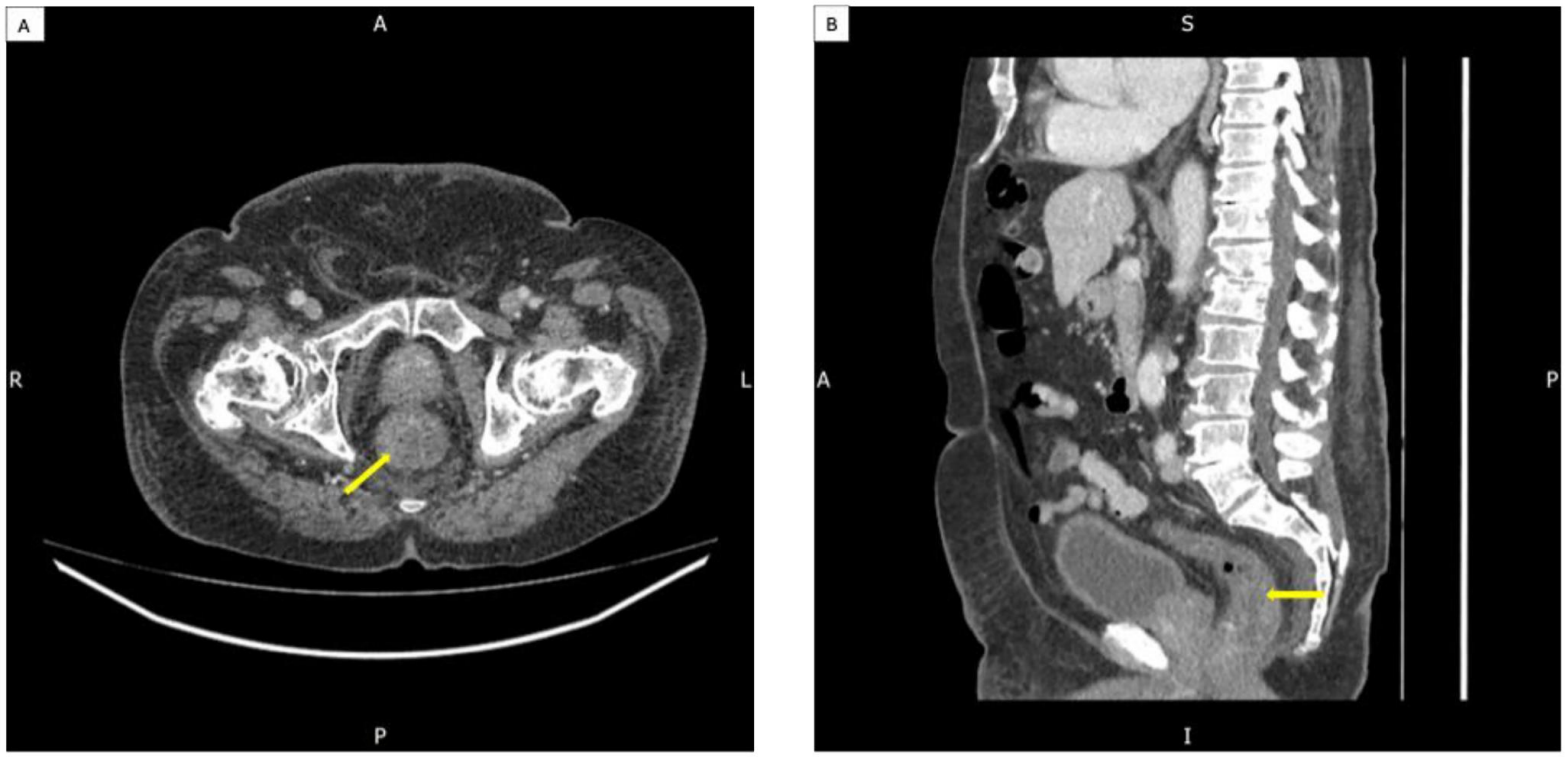
Diffuse circumferential thickening of the rectum with luminal obliteration, which initially raised the possibility of a malignant cause, was later thought to represent proctitis. Axial image of the rectum (A); sagittal view of the rectum (B).

Whilst awaiting transthoracic echocardiography to exclude infective endocarditis, the patient developed rectal bleeding. This prompted a request for a CT scan of the thorax, abdomen, and pelvis to investigate potential colorectal malignancy and to identify the source of the *E. coli* bacteraemia. The CT scan report suggested circumferential rectal thickening, raising suspicion of a rectal tumour. The case was discussed in a colorectal multidisciplinary team (MDT) meeting, where it was deemed a probable case of proctitis (Figure 1).

**Figure 2.**
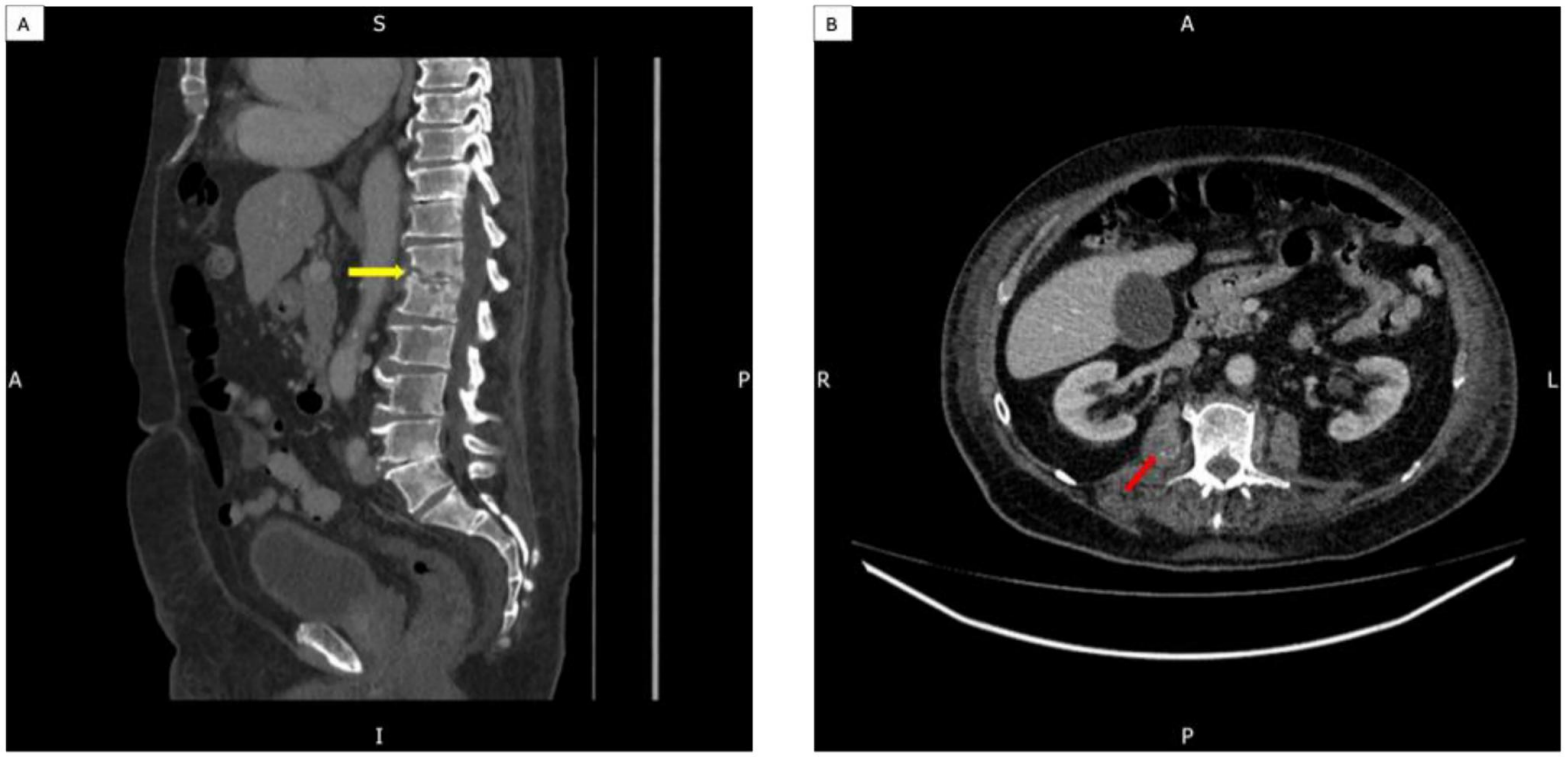
Bony changes in the endplates of T12/L1, with partial collapse of the end plates and lytic changes, were noted close to the disc level. Image A shows bone changes with mixed sclerosis and lytic changes more in keeping with spondylodiscitis; image B shows a bulky right psoas muscle with low attenuation changes suggestive of a small psoas abscess.

The CT scan also revealed a right-sided psoas abscess and spondylodiscitis at T12 and L1 (Figure 2).

**Figure 3.**
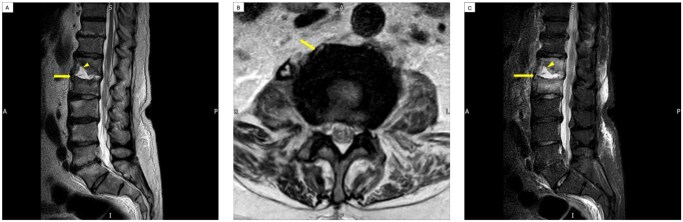
Sagittal T2 and axial T2 weighted images (A, B) and sagittal STIR sequences (C) from subsequent MRI lumbar spine showing T12/L1 disc liquefaction with partial collapse of the inferior endplate of T12/L1, small anterior erosions and marked bone marrow oedema.

Following consultation with an interventional radiologist, the psoas abscess was deemed unsuitable for drainage. Subsequent MRI of the thoracic and lumbar spine showed disc liquefaction at T12/L1 with small paravertebral collections extending into the right psoas muscle (Figure 3).

**Figure 4.**
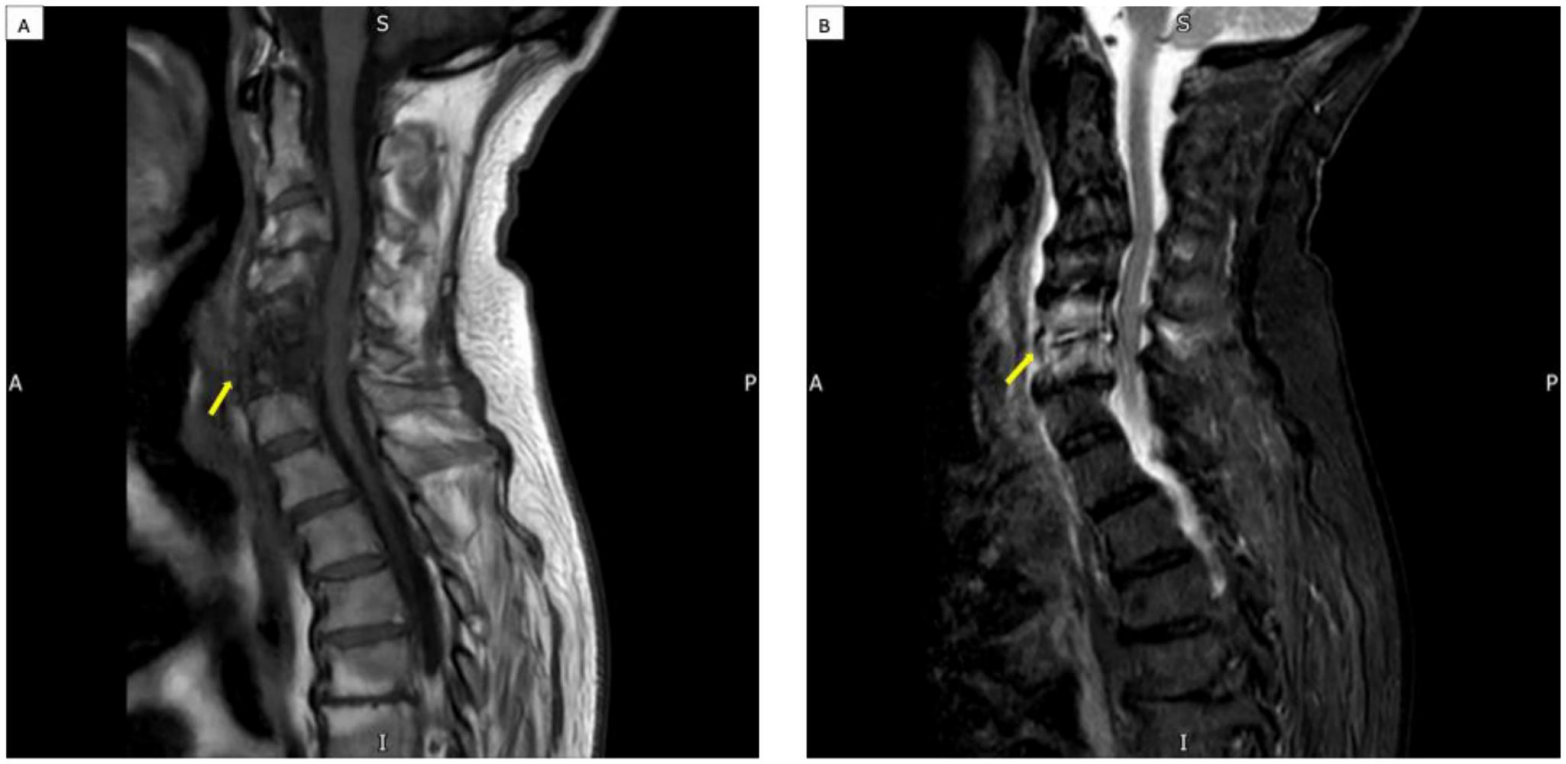
MRI cervical spine T1-weighted image (A) and sagittal STIR (B) showing bone marrow oedema and end-plate changes at C5/6 with a small abscess also developing at this level due to a second level of spondylodiscitis (axial sequences not shown).

Another focus of spondylodiscitis was demonstrated at C5/C6. A dedicated MRI of the cervical spine confirmed infective spondylodiscitis at C5/C6, involving the intervertebral disc, adjacent endplates, bilateral facet joints, and a possible small anterior prevertebral abscess. Mild canal stenosis was observed due to chronic degenerative spondylosis, but no intrinsic myelopathy was present (Figure 4).

Antibiotic therapy was escalated to intravenous piperacillin and tazobactam based on bacterial sensitivity to treat both the psoas abscess and infective discitis.

## Other investigations

Viral screening, including an HIV test, was negative. Multiple myeloma was excluded, with normal calcium levels and a polyclonal increase in immunoglobulins. Transthoracic and subsequent transoesophageal echocardiography were performed after neurosurgical consultation. There was no evidence of infective endocarditis. The neurosurgical team advised that there was no spinal instability and that surgical intervention was not required. Flexible sigmoidoscopy showed no evidence of malignancy or inflammation.

## Subsequent management, outcome, and follow-up

Despite treatment, the patient experienced persistent fever spikes, and repeated blood cultures continued to show *E. coli* bacteraemia. Subsequent urine cultures identified an extended-spectrum beta-lactamase (ESBL)-producing *E. coli* and *Enterococcus faecalis*, complicating treatment in this elderly patient with multiple co-morbidities. Infectious disease specialists and microbiologists were consulted. The antibiotic regimen was switched to intravenous meropenem. Long-term antibiotic therapy was tentatively planned for 6 weeks. The suspected sources of *E. coli* bacteraemia included colorectal, urinary, and spinal infection; the latter being more supported by the imaging findings.

The patient showed clinical improvement with medical management, avoiding surgical debridement, interventional aspiration, or drainage. Biochemical markers normalized, and the multi-focal spondylodiscitis was successfully managed. This can be attributed to the collaborative efforts of a multidisciplinary team, including infectious disease specialists, microbiologists, radiologists, oncologists, cardiologists, and neurosurgeons. An outpatient antibiotic treatment (OPAT) plan was established to ensure an effective long-term antibiotic regimen to allow the patient to be discharged and managed at home. At his last clinic visit and follow-up blood tests, no evidence of an ongoing infection was found, and the patient was discharged from the infectious disease team upon completion of the antibiotics. A follow-up colonoscopy has been scheduled to remove polyps found on the previous inpatient sigmoidoscopy.

## Discussion

Spinal infection is not uncommon, with an annual incidence of approximately 2.2 per 100,000 individuals.^1^ In England, spondylodiscitis admissions increased by 33% from 2012 to 2021, with the most significant rises observed in individuals aged 70-74 (117%) and 75-79 (133%).^2^ Psoas abscesses occur in about 12% of spondylodiscitis cases.^3^ The lumbar region is most frequently affected, while cervical infections are more commonly observed among intravenous drug users. Spondylodiscitis can be categorized into three primary types: granulomatous, parasitic, and pyogenic infections.^2^ It can arise through three primary mechanisms: haematogenous dissemination from a distant infection site, direct inoculation following trauma (such as injury or surgery), and spread from contiguous tissues.^1^ Granulomatous spondylodiscitis encompasses infections caused by *Mycobacterium tuberculosis*, *Brucella* spp., fungi, and *Aspergillus* spp. Pyogenic spondylodiscitis, the most common variant in the Western world, is predominantly attributed to *Staphylococcus aureus.*^2^  *Escherichia coli* is a rare aetiological agent of pyogenic vertebral infections, contributing to approximately 11%-25% of cases.^4^ Risk factors for *E. coli* spinal infections include advanced age, immunosuppression, malignancy, diabetes mellitus, long-term corticosteroid use, malnutrition, untreated prostatitis, intravenous drug use, and recent neurosurgical procedures.^5^ These factors contribute to an increased susceptibility to *E. coli* infections in the spinal region, underscoring the need for careful consideration of such risks in both diagnosis and management.

Diagnosing spondylodiscitis involves integrating clinical presentation, laboratory results, and radiological imaging.^6^ MRI is the preferred imaging method for evaluation due to its excellent ability to visualize soft tissue pathologies and neural structures. It is essential for confirming spondylodiscitis and detecting complications such as spinal cord compression and differentiating from malignancies.^6^^,^^7^ However, MRI has limitations, including potential artefacts from spinal hardware, incompatibility with metal implants, limited availability in some settings, and delayed assessment of early treatment responses.^6^ These factors, combined with similar MRI findings in erosive osteochondritis with Modic Type 1 changes and compression fractures, can complicate the accurate diagnosis of early-stage spondylodiscitis.^8^

When MRI is not feasible or suitable, radionuclide imaging provides a valuable alternative. Among these, Fluorine-18 fluorodeoxyglucose positron emission tomography (^18^FDG-PET or PET/CT) is particularly effective for diagnosing spondylodiscitis.^6^^,^^7^ It can distinguish between degenerative (Modic type I) and infectious vertebral abnormalities. It is also useful in patients with spinal hardware, aiding in monitoring treatment response and postoperative evaluation.^6^^,^^9^

Treatment for spondylodiscitis involves conservative methods, including antimicrobial therapy, physiotherapy, and immobilization, as well as surgical intervention for neural compression and spinal instability. Interventional radiology may also be employed to drain paraspinal abscesses where suitable.^7^

In elderly patients with multiple co-morbidities, diagnosis and management could be challenging. This is because the source of infection can be masked, particularly in those with polymicrobial infections or those with resistant organisms. In our case of an elderly patient, the initial presentation was fever and lower back pain with the background history of prostate cancer and valvular heart disease. It made the differential diagnosis more complex, necessitating a thorough appraisal of the clinical, microbiological, and imaging investigations. The identification of *E. coli* in blood cultures, without an immediately apparent source, poses a diagnostic dilemma, since it is the most typical infection for genitourinary, gastrointestinal and colorectal in origin.

The development of rectal bleeding initially complicated the clinical picture, suggestive of rectal malignancy or a gastrointestinal source of origin. When investigating the rectal bleeding, a significant diagnostic turning point was the detection of a right psoas abscess and spondylodiscitis at multiple spinal levels on CT. This was also mitigated by multidisciplinary discussion, which suggested a diagnosis of proctitis rather than neoplastic disease.

Another challenging radiological sign was perinephric stranding. The presence of perinephric fat stranding in this case also raised the question of whether an upper urinary tract infection might have disseminated, causing discitis. It is usually a non-specific sign on imaging for pyelonephritis. It can also be present in other conditions, such as renal dysfunction or collecting system dilatation. The sign may also be secondary to adjacent spondylodiscitis and the development of a psoas abscess.

Additionally, the patient’s aortic valvular disease could also have been the source of systemic infection origin in the context of bacteraemia. However, *E. coli* is a very rare organism to cause native valve endocarditis. However, it can still occur in the elderly, diabetics and those on immunosuppressive therapy or progressive cancer.

The emergence of ESBL-producing *E. coli* and *Enterococcus faecalis* in subsequent urine cultures added another layer of complexity to the case. ESBL-producing organisms are increasingly prevalent and pose significant treatment challenges due to their resistance to many standard antibiotics. The patient’s prior exposure to co-trimoxazole and subsequent switch to ciprofloxacin likely influenced the microbiological landscape. It is also important to highlight the need for antimicrobial stewardship and careful selection of empiric therapy in patients with recurrent or resistant infections. Management included transition to intravenous meropenem, reflecting the necessity of using broader-spectrum antibiotics in the face of resistant pathogens.

The successful resolution of a spinal infection in our case underscores the critical role of a MDT in achieving optimal patient outcomes. This collaborative approach addresses the challenges of diagnosing infection in multi-morbid elderly patients and the complexities of managing resistant organisms without surgery.

## Learning points

- Consider *Escherichia coli* (*E. coli*) as a cause of multi-level spondylodiscitis in elderly patients with recurrent bacteraemia, even when a genitourinary cause is not apparent.
- Radiological investigations, specifically MRI, are crucial for diagnosing *E. coli* spondylodiscitis but may be underutilized, emphasizing the need for thorough evaluation in patients with unexplained back pain and bacteraemia.
- Maintaining a high index of suspicion for *E. coli*-associated spondylodiscitis is essential, particularly in at-risk populations, to ensure early investigation and timely management.
- Early and appropriate intervention is vital to prevent serious complications, including neurological deficits.
- The role of multidisciplinary care is critical in managing complex medical cases.

## Informed consent statement

Informed consent was obtained from the patient to publish this case report and accompanying images.

## Conflicts of interest

None declared.
